# Aromatherapy Benefits Autonomic Nervous System Regulation for Elementary School Faculty in Taiwan

**DOI:** 10.1155/2011/946537

**Published:** 2011-04-10

**Authors:** Kang-Ming Chang, Chuh-Wei Shen

**Affiliations:** ^1^Department of Photonics and Communication Engineering, Asia University, Taichung 41354, Taiwan; ^2^Graduate Institute of Clinical Medical Science, China Medical University, Taichung 40402, Taiwan; ^3^Ming-Chien Elementary School of Nantou County, No. 220, Jhangnan Rd., Ming-Chien Township, Nantou County 55146, Taiwan

## Abstract

Workplace stress-related illness is a serious issue, and consequently many stress reduction methods have been investigated. Aromatherapy is especially for populations that work under high stress. Elementary school teachers are a high-stress working population in Taiwan. In this study, fifty-four elementary school teachers were recruited to evaluate aromatherapy performance on stress reduction. Bergamot essential oil was used for aromatherapy spray for 10 minutes. Blood pressure and autonomic nervous system parameters were recorded 5 minutes before and after the application of the aroma spray. Results showed that there were significant decreases in blood pressure, heart rate, LF power percentage, and LF/HF while there were increases in heart rate variability and HF power percentage (*P* < .001^∗∗∗^) after application of the aromatherapy spray. Further analysis was investigated by dividing subjects into three background variables (position variables, age variables, gender variables) and anxiety degree groups. All parameters were significantly different for most subgroups, except for the substitute teachers and the light-anxiety group. Parasympathetic nervous system activation was measured after aromatherapy in this study. It encouraged further study for other stress working population by aromatherapy.

## 1. Introduction

Workplace stress has attracted much attention recently [1]. In Taiwan, working stress is increasing among elementary school teachers, as a result of students declining, competition between schools, and education reform. Local studies have indicated that there is higher job stress and interpersonal stress in male elementary teachers than in female teachers. Young teachers feel increased stress on income and time scheduling; while senior teachers feel increased stress on colleague relationships [2]. Thus, appropriate stress coping methods are also highly desirable.

Various approaches have been involved in workplace stress management [3]. Aromatherapy, due to easy implementation and effectiveness, is one of them [4]. Essential oils are used to reduce body tension and emotional stress. The most common types of essential oils are bergamot, lavender, and geranium [5]. Use of lavender and rosemary scented candles has been found to reduce the test pressure of nursing school students [6]. Aromatherapy was widely used for stress adaptation. Komarova and Avilov's results showed that regular use of fragrant scented candles can increase students' parasympathetic rhythm [7]. Seo thought inhalation aromatherapy was an effective stress management method. Their study included 36 female high school students, and stress levels were significantly lower when the students received the aroma treatment [8]. In addition, Hur et al. applied aromatherapy massage in Korean climacteric women. Lavender rose geranium, rose, and jasmine in almond and primrose oils were used for massage once a week. Eight-week massage showed a significantly lower total menopausal index than that in wait-listed controls. These findings suggest that aromatherapy massage can be an effective treatment of menopausal symptoms such as hot flushes, depression, and pain in climacteric women [9]. Bagetta et al. examined the brain wave spectrum power and found that bergamot essential oil correlate well with its exocytotic and carrier-mediated release of discrete amino acids endowed with neurotransmitter function in the mammalian hippocampus. Bergamot essential oil was able to interfere with normal and pathological synaptic plasticity. Therefore, Bergamot essential oil was effective for anxiety reduction of mild depression subjects, and it also had the effect of reducing pain in cancer subjects [10].

Therefore, bergamot essential oil is used in this study, with the aid of a sprayer for aromatherapy. Many essential oils have sedating effect, such as bergamot, lavender, chamomile, and other essential oils [5]. However, chamomile was at a high price, and lavender odor was irritating and allergic to part of the subjects. Therefore, bergamot essential oil was chosen in this study.

The autonomic nervous system includes sympathetic activity and parasympathetic activity. When people feel anxious or experience stress, heart rate, and sympathetic activity will increase, together with decreasing parasympathetic activity. Narita et al. found that there were higher sympathetic activities for depressed and anxious subjects than for normal subjects [11]. Similar results were also shown on swimmers [12]. Autonomic nervous system activities can be monitored from heart rate variability, which was derived from heartbeat interval time series. With further discrete Fourier transform applied to the heartbeat interval time series, two specific power spectrum ranges were defined. One is low frequency range (LF, 0.04–0.15 Hz) and the other is high frequency range (HF, 0.15–0.4 Hz).

 Therefore, significant HRV variation was expected after aromatherapy. Sympathetic activity is accompanied with increases in low frequency power (LF) of the heart rate variability (HRV) spectrum, while parasympathetic activity is associated with high frequency power (HF). Duan et al.'s results indicated that after the inhalation of lavender, there was a significant increase in HF and LF/HF values [13]. Anxiety caused by edited films was reduced with the aid of lavender aromatherapy, and there was an increase in HRV [14]. Therefore HRV was used as an indicator to measure the effect of aromatherapy on elementary school teachers.

## 2. Material and Methods

### 2.1. Subjects

Fifty-four elementary school teachers from three different schools were enrolled. Possible asthma, hypertension, or heart disease patients were excluded. The reason to exclude asthma is that aromatherapy spray may induce asthma, so the items excluded in this experimental study on asthma patients. Furthermore, many studies indicate that heart rate variability is closely related to heart disease, myocardial infarction, and heart failure, so the subjects with heart disease and hypertension are also excluded from this study. 

Further subgroups were divided by gender, position, age, and anxiety degree. The Beck Anxiety Inventory (BAI) was used by qualified expert to estimate the degree of anxiety in each volunteer. Based on the BAI result, subjects were scored as light anxiety, mild anxiety, or moderate anxiety. Detailed subject information is listed in Table 1.

### 2.2. Experimental Procedures

Aromatherapy was conducted once a week. Physiological recordings were taken during the second week in the school's health center. An Ultrasonic Ionizer Aromatherapy Diffuser was used for aroma evaporation (type YHL668/I, ultrasound frequency 2.5 MHz, Nature Creart Co. Ltd, made in Taiwan). 100% pure bergamot essential oil was used and diluted to 2%. Physiological parameters were recorded by an ANSWatch monitor (TS-0411 type, Taiwan Scientific Ltd., which has been approved by ISO 13485, and EU CE Mark). Each session was recorded for seven minutes. Average blood pressure and HRV parameters were shown on the panel of ANSWatch monitor. Detailed experimental procedures were as follows. IRB was approved by Asia University Medical Research Ethics committee.

**Figure 1:**
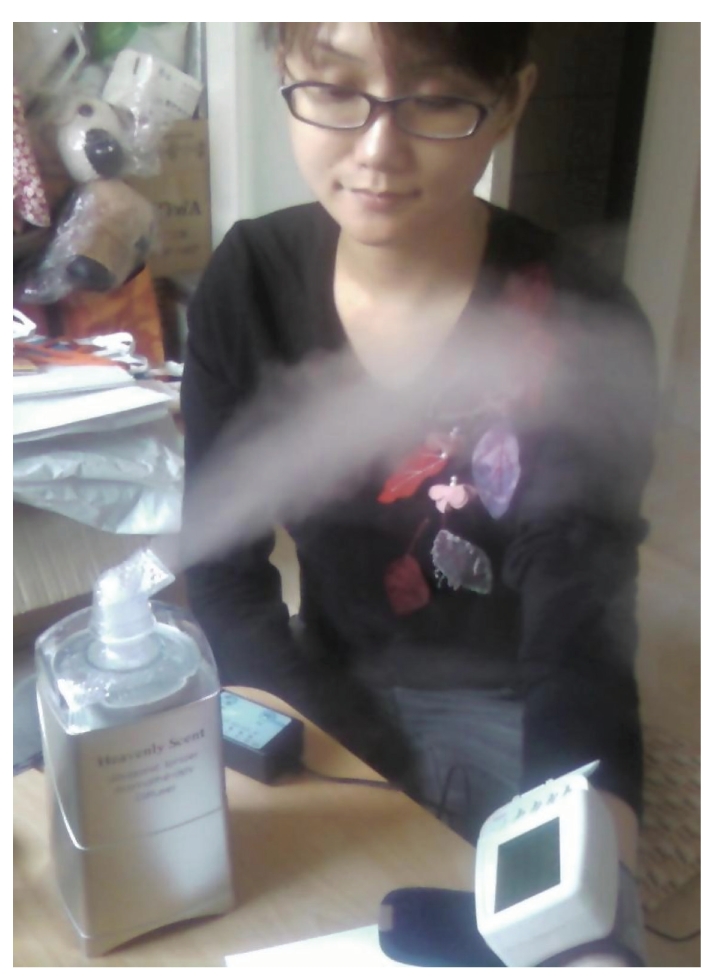
Illustration of aromatherapy for elementary school teacher.

- Basic subject information was collected (height, weight, BMI, age range). Each subject was required to fill in a consent form and a BAI survey. First and second aromatherapies were conducted on the same weekday and at the same time. Smoking, alcohol drinking, and coffee were forbidden six hours before aromatherapy.
- Subjects were asked to rest for five to ten minutes before HRV recording. Then, a pretest recording of 7 minutes was made (sitting, eyes open, not doing any activity).
- Ten minutes aromatherapy intervention was conducted by the same nurse, as demonstrated in Figure 1. Respiration rate and respiration volume in this session were required the same as that during rest period.
- Posttest recording for another 7 minutes.

### 2.3. Physiological Parameter Collection

ANSWatch monitor records two blood pressure parameters and five HRV parameters, as shown below.

- SYS (mmHg): systolic blood pressure.
- DIA (mmHg): diastolic blood pressure.
- HR (BPM): average heart beat in terms of beats per minutes (BPM).
- HRV (ms): heart rate variability, a similar term to SDNN, defined as standard derivation of RR interval sequence.
- HF (%): high frequency power percentage. HF frequency range was between 0.15–0.4 Hz on heart rate variability spectrum.
- LF (%): low frequency power percentage. LF frequency range was between 0.04–0.15 Hz on heart rate variability spectrum.
- LF/HF: ratio of LF power to HF power.

### 2.4. Statistics

In this study, the SPSS 12.0 software package was used to conduct data analysis. Significance test for the alpha value was set at 0.05. Several statistical methods were used, as follows.

- Descriptive statistics: personal information on subjects is represented as mean ± standard deviation (mean ± SD).
- Paired *t*-test: intragroup differences among three background variables “gender,” “age” and “position” were compared. Seven parameters within each group, SYS, DIA, HR, HRV, HF%, LF%, and LF/HF were examined.
- Analysis of Covariance (ANCOVA): pretest was used as covariates, intergroup difference among position, age, gender, and anxiety degree was examined by ANCOVA. The Scheffe method and the Post-hoc test for least significance difference test were used for post-hoc test. A three-way ANCOVA was also used to examine the interaction effect among the three background variables “gender,” “age” and “position.”

## 3. Results

Paired *t*-test results for Bergamot essential oil treatments for all subjects are listed in Table 2. According to Table 2, it is apparent that after the aromatherapy treatment blood pressure is reduced, both on SYS and on DIA. There was also a decrease in heart rate, LF% and LF/HF. Apparently, treatment of aromatherapy increases parasympathetic nervous activity; therefore, HF and HRV parameters will increase.

Further analysis of subgroups is shown in the following. The position group result is shown in Table 3. Similar to Table 2, there were significant differences on all physiological parameters for administrative staff and for homeroom teachers. Although there was also a significant reduction in blood pressure and heart rate, there was not enough statistical difference on HF% and LF% for the substitute teachers. According to further in-depth interviews with substitute teachers, that may be associated with the coming annual faculty entrance test. Substitute teachers had to prepare for the test in their spare time, they cannot relax and, therefore, aromatherapy's performance is reduced. 

Subgroup results for age and gender are listed in Tables 4 and 5, respectively. There were significant differences due to aromatherapy for all subgroup on all physiological parameters. The subgroup with anxiety is also tested and shown in Table 6. The high anxiety and moderate anxiety groups had similar results to those shown in Table 2. Aromatherapy was effective for high anxiety and moderate anxiety groups. The light anxiety group had the same performance as the substitute teacher group; there was no statistical difference on HF% and LF%, but there were significant differences for the other five parameters. After further checking of population distribution, there was no population overlap between the light anxiety group and the substitutive teacher group. The light anxiety was not fully affected by aromatherapy. A possible reason may be the stable autonomic nervous system for the light anxiety group. Therefore, there was no further activation of parasympathetic activity.

Analysis of covariance between age and position groups was evaluated. There was no significant difference for all seven physiological parameters. Similar results were also shown for the covariance analysis between gender and anxiety degree groups. In other words, aromatherapy performance was similar for all these groups.

## 4. Discussion

High workplace stress is an important personal health risk factor; it is also harmful for an enterprise's benefits. The enterprise's benefit was with healthier employees that would reduce the extra cost due to high stress employees and support better education service for students. Elementary school teachers are chosen in this study as the experimental groups due to being a high work-stress group. In this study, physiological signals were measured after the second once-weekly aromatherapy treatment. This experiment focused on the short-term stress relaxing effect instead of tracking the long-term effect of aromatherapy. Data showed that that aromatherapy would be effective in promoting parasympathetic activation, reducing blood pressure and heart rate. Therefore, aromatherapy may be useful to provide relief from working stress. Further subgroup analysis revealed that aromatherapy was also effective on groups arranged by gender, age, and positions.

The personal characteristics that were found in association with aromatherapy performance include degree of anxiety measured using the BAI. This study also found that aromatherapy was effective for moderate to severe anxiety groups. However, there was no significant statistical effect for the light anxiety group, which was stable for autonomic nervous activity. The function of aromatherapy is to drive the autonomic nervous activity toward a balanced state; therefore, there was limited physiological change after aromatherapy treatment. Whether aromatherapy is beneficial for long-term anxiety reduction is still an open issue. 

Bergamot essential oil is a good choice for aromatherapy, although there are many studies using lavender oil. Lavender oil was not chosen due to some subject's report of allergy to lavender oil. Bergamot essential oil is more moderate for users and is a lower price. In the future, aroma within air-conditioning will be beneficial to create a better working environment.

Many studies have confirmed that aromatherapy was useful for stress reduction, which is not only limited to the spray inhalation way, but also used by transdermal application experiment [15, 16]. This study further investigated aromatherapy on different variables on faculty, such as gender, age, position, and level of anxiety. The control group with water spray as a placebo was expected in the future study. The other factor that may affect aromatherapy performance is the odor preferences of different individuals. Subjects' personal likes or dislikes could lead to different results, but this factor was not considered in this study. However, it could be interesting to be examined in the further experiment.

## 5. Conclusion

After two 10-minute aromatherapy sprays with Bergamot essential oil on elementary school teachers, the parasympathetic nervous system was enhanced and shown on corresponding physiological parameters. Aromatherapy seems to drive autonomic nervous activity toward a balanced state. Subjects with moderate and high degrees of anxiety benefited more than the light anxiety group. With strict control of experimental environment, including subject's posture, measurement location and time, and experimental procedures, this study rules out many possible factors that affect the body's physiological signals. This article provides useful information about aromatherapy stress reduction performance on different faculty groups. It encouraged further study for other stress working population by aromatherapy.

## Figures and Tables

**Table 1:** Subject information (*n* = 54).

Position	Administrative staff (*n* = 19)
	Homeroom teacher (*n* = 21)
	Substitute teacher (*n* = 14)
Gender	Male (*n* = 25)
	Female (*n* = 29)

Age	Below 34 (*n* = 21)
	35–44 (*n* = 19)
	Above 45 (*n* = 14)

Anxiety degree	Light (*n* = 26)
	Mild (*n* = 17)
	Moderate (*n* = 11)

**Table 2:** Paired *t*-test result for aroma for all subjects.

Items	All	*P* value
SYS (mmHg)		
Before	123.30 ± 12.810	.001***
After	112.78 ± 15.909	

DIA (mmHg)		
Before	82.91 ± 7.86	.000***
After	76.76 ± 7.997	

HR (BPM)		
Before	83.15 ± 13.964	.000***
After	74.61 ± 9.803	

HRV (ms)		
Before	137.54 ± 69.215	.000***
After	197.89 ± 91.195	

HF (%)		
Before	50.93 ± 15.331	.004**
After	61.13 ± 10.622	

LF (%)		
	49.57 ± 15.320	.004**
	38.37 ± 10.010	

LF/HF		
Before	1.170 ± 0.8348	.005**
After	0.648 ± 0.2800	

**Table 3:** Position subgroup paired *t*-test result for aroma.

Items	Administrative staff (*n* = 19)	Homeroom teacher (*n* = 21)	Substitute teacher (*n* = 14)
SYS (mmHg)			
Before	123.16 ± 11.34	123.10 ± 14.74*	123.79 ± 12.50**
After	111.16 ± 15.74	113.43 ± 17.10	114.00 ± 15.27

DIA (mmHg)			
Before	82.37 ± 7.32**	82.00 ± 8.78**	85.00 ± 7.26**
After	76.68 ± 9.05	76.10 ± 7.91	77.86 ± 7.02

HR (BPM)			
Before	83.42 ± 12.37**	84.71 ± 17.78*	80.43 ± 9.21**
After	73.42 ± 9.71	76.57 ± 10.34	73.29 ± 9.29

HRV (ms)			
Before	138.84 ± 79.54**	147.38 ± 63.93**	121.00 ± 63.48**
After	216.26 ± 108.82**	205.67 ± 71.53	161.29 ± 87.21

HF (%)			
Before	48.95 ± 16.54**	52.86 ± 12.23*	50.71 ± 18.37
After	64.58 ± 6.19	60.10 ± 8.01	59.07 ± 16.04

LF (%)			
Before	51.05 ± 16.54**	47.14 ± 12.23*	49.29 ± 18.37
After	35.42 ± 6.19	39.90 ± 8.01	40.93 ± 16.04

LF/HF			
Before	1.33 ± .99**	0.99 ± 0.48*	1.22 ± 1.02*
After	0.56 ± 0.146	0.71 ± 0.25	0.72 ± 0.48

*P < .05; **P < .01; ***P < .001.

**Table 4:** Age subgroup paired *t*-test result for aroma.

Items	Below 34 (*n* = 21)	35–44 (*n* = 19)	Above 45 (*n* = 14)
SYS (mmHg)			
Before	124.43 ± 10.06**	119.32 ± 15.26*	127.00 ± 12.29**
After	114.24 ± 11.49	109.26 ± 20.99	115.36 ± 13.78

DIA (mmHg)			
Before	82.62 ± 7.18**	81.11 ± 7.26**	85.79 ± 9.26**
After	77.48 ± 6.19	73.95 ± 7.95	79.50 ± 9.69

HR (BPM)			
Before	80.71 ± 10.52**	87.42 ± 18.04*	81.00 ± 11.52**
After	72.29 ± 8.36	77.00 ± 11.16	74.86 ± 9.74

HRV (ms)			
Before	131.86 ± 70.25**	172.37 ± 72.99**	98.79 ± 33.48**
After	190.10 ± 98.56	232.84 ± 97.32	162.14 ± 50.86

HF (%)			
Before	51.52 ± 16.46*	51.89 ± 15.51*	48.71 ± 14.18*
After	62.33 ± 10.03	60.95 ± 11.22	61.36 ± 8.64

LF (%)			
Before	48.48 ± 16.46*	48.11 ± 15.51*	51.29 ± 14.18*
After	37.67 ± 10.03	39.05 ± 11.22	38.64 ± 8.64

LF/HF			
Before	1.200 ± 0.87**	1.12 ± 0.962*	1.200 ± 0.618**
After	0.66 ± 0.35	0.62 ± 0.21	0.679 ± 0.27

*P < .05; **P < .01; ***P < .001.

**Table 5:** Gender subgroup paired *t*-test result for aroma.

Items	Male (*n* = 25)	Female (*n* = 29)
SYS (mmHg)		
Before	124.52 ± 11.31**	122.24 ± 14.086**
After	114.04 ± 17.29	111.69 ± 14.837

DIA (mmHg)		
Before	82.00 ± 6.89**	83.69 ± 8.652**
After	77.32 ± 8.72	76.28 ± 7.440

HR (BPM)		
Before	81.28 ± 11.50**	84.76 ± 15.813**
After	73.24 ± 8.82	75.79 ± 10.584

HRV (ms)		
Before	124.08 ± 71.86**	149.14 ± 65.883**
After	191.56 ± 102.44	203.34 ± 81.740

HF (%)		
Before	49.12 ± 17.74**	53.28 ± 12.029**
After	61.08 ± 11.84	61.24 ± 8.967

LF (%)		
Before	50.88 ± 17.74**	46.72 ± 12.029**
After	38.92 ± 11.84	38.76 ± 8.967

LF/HF		
Before	1.30 ± 1.017**	0.993 ± 0.5451**
After	0.66 ± 0.35	0.672 ± 0.2658

*P < .05; **P < .01; ***P < .001.

**Table 6:** Anxiety degree subgroup paired *t*-test result for aroma.

Items	Light (*n* = 26)	Mild (*n* = 17)	Moderate (*n* = 11)
SYS (mmHg)			
Before	119.19 ± 11.90*	121.94 ± 13.08**	135.09 ± 6.49*
After	110.19 ± 18.84	111.88 ± 12.47	120.27 ± 11.16

DIA (mmHg)			
Before	82.92 ± 7.63**	80.88 ± 8.63**	86.00 ± 6.72*
After	76.73 ± 8.19	75.47 ± 7.238	78.82 ± 8.94

HR (BPM)			
Before	85.77 ± 16.22**	81.82 ± 13.12**	79.00 ± 7.77*
After	76.19 ± 10.25	74.00 ± 9.507	71.82 ± 9.30

HRV (ms)			
Before	160.19 ± 64.25**	112.65 ± 71.38**	122.45 ± 65.42**
After	222.65 ± 95.88	178.47 ± 85.98	169.36 ± 78.34

HF (%)			
Before	54.19 ± 17.28	49.94 ± 11.64**	44.73 ± 14.52**
After	61.35 ± 10.96	63.41 ± 6.90	59.36 ± 11.80

LF (%)			
Before	45.81 ± 17.28	50.06 ± 11.64**	55.27 ± 14.52**
After	38.65 ± 10.96	36.59 ± 6.90	40.64 ± 11.80

LF/HF			
Before	1.09 ± 1.02*	1.13 ± 0.58**	1.418 ± 0.67**
After	0.627 ± 0.23	0.61 ± 0. 18	0.764 ± 0.45

*P < .05; **P < .01; ***P < .001.
